# What ICU nurses in different Austrian hospitals know and think about the Austrian organ donation law

**DOI:** 10.1186/1472-6939-15-46

**Published:** 2014-06-17

**Authors:** Gabriele Zettel, Angela Horvath, Ekaterina Vorobyeva, Christian Auburger, Michael Zink, Philipp Stiegler, Vanessa Stadlbauer

**Affiliations:** 1 Department of Surgery, Division of Transplantation Surgery, Medical University of Graz, Auenbruggerplatz 21, 8036 Graz, Austria; 2 Department of Internal Medicine, Division of Gastroenterology and Hepatology, Medical University of Graz, Graz, Austria; 3 General Public Hospital of the Order of Saint Elisabeth, Linz, Austria; 4 Brothers of St. John of God Hospital, St. Veit/ Glan, Austria

## Abstract

We previously reported a high level of information on the Austrian organ donation law in medical and non-medical students, patients and ICU nurses, whereby ICU nurses at University Hospital in Graz (n = 185) were very well informed and also had the most critical view of the Austrian organ donation law.

This letter reports the extension of our previous study to ICU nurses from hospitals with a Christian background (n = 60). We found that ICU nurses in hospitals run by religious congregations considered the Austrian organ donation law to be good more often than did those at the University Hospital in Graz. A positive attitude was also influenced by gender and prior knowledge of the law.

Reasons for this could be the Christian orientation of the hospitals or exposure to organ donation and transplantation procedures on the job.

## 

Organ transplantation is an important therapy option in many end-stage diseases but mortality for patients on the waiting lists ranges from 3-42% due to the great demand for donor organs. Austria has a relatively high donation rate of 22.7 donors per one million inhabitants [1]. The legislation in Austria may be a reason for the relatively high number of organ donors and resultantly shorter waiting times than in other countries. Since 1982, the Austrian law has provided an “opt-out” option for organ donation. A person who does not want to be an organ donor in the case of brain death must declare this wish in advance, for example by putting his/her name on the contradiction registry [2]. About 0.25% of the Austrian population are registered.

In 2013 we reported high level of information on the organ donation law in students, patients and ICU nurses, whereby ICU nurses at University Hospital in Graz were very well informed and also had the most critical view of the Austrian organ donation law. Low acceptance was most prominent in women between 31 and 40 years of age despite their high level of information, although more information has been shown to increase acceptance in students in health related and unrelated studies [3].

To further examine reasons for this critical view we conducted a similar survey in some other ICUs in Austria. All the additional units are run by organizations with a Christian background that expect their staff to adhere to Christian values in their work. Nurses from the University Hospital in Graz (n = 185) and from Austrian hospitals run by religious congregations (n = 60) were compared. The study was approved by the institutional review board of the Medical University of Graz (EK 24–140 ex 11/12). Participants taking the online survey were informed about the purpose, the investigators, the length of the survey, and the anonymity of the stored data. Participation in the survey implied informed consent for the data analysis. Participants were asked to fill in the questionnaire containing questions about demographic data (sex, age group, education level). Having answered these questions, the participants received information about the necessity of organ transplantations in general and the organ donation legislation in Austria (see Additional file 1 for details) as an integral part of the online survey or as information leaflet. After having read the information, the participants were asked if they already knew the Austrian law before having read the information part of the survey. The following 2 questions focused on the opinions and attitudes towards the law and on the changes of their attitude after having received detailed information about the law. The question measuring opinions and attitudes gave 4 possibilities where the participants were asked to pick either one or more most adequate answers presenting their opinion. The final “shift of opinion” question offered 4 possibilities with single choice to answer. The questionnaire and the information given to the participants are given as Additional file 1.

The two groups were comparable in age, education and knowledge of the law. Women were overrepresented at the University Hospital in Graz compared to Christian hospitals (89.7% vs. 71.6%, respectively). There was no difference between the hospitals regarding the questions whether the law should stay as it is or if an active donor list would be preferable or if the participants plan or consider to put their names on the contradiction registry.

The nursing staff of the University Hospital in Graz views the law more critically. There, the legislation was thought to be a good by 58.9% of participants, significantly less than in Christian hospitals (76.6%, p = 0.013). Regardless of their hospital affiliation, women considered the law “good” less often than men (60.8% vs. 77.8%, respectively, p = 0.051). The differences between the hospitals however were not accountable to the gender differences (Figure 1A).

**Figure 1:**
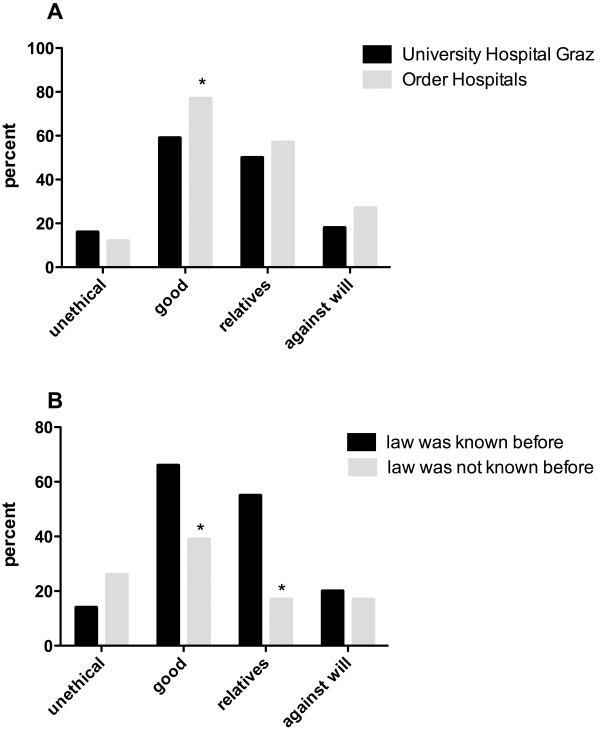
**Opinion of ICU nurses on the Austrian organ donation law. A**: Opinion of ICU nurses at the university hospital in Graz compared to Christian Hospitals on the Austrian organ donation law. **B**: Opinion of ICU nurses towards the Austrian organ donation law depending on their information level (law previously known or not). Legend: Unethical: The law cannot be ethically justified; it is unethical, as every human being should be able to decide for him/herself whether or not to donate organs. An active donation register should be introduced. Good: The Austrian legislation is good, as more patients on waiting lists can be helped. Relatives: It is important to consider and accept the opinions of relatives, although the donation rates might decrease. Against will: Provided that potential organ donors did not choose the opt-out option during their lifetimes, it should be possible to retrieve their organs against the will of the relatives, as the intention of the deceased person can no longer be ascertained. As multiple answers were permitted for this question, the sum of the answers is more than 100%.

Age, education and training did not influence opinion on the law but knowledge of the law prior to the survey did. Nurses who were familiar with the law before the survey classified it as “good” more often than did nurses without prior knowledge (65.8% vs. 39.1%, respectively, p = 0.012). Informed staff voted more often for considering the wishes of the donor’s family than uninformed participants (55.0% vs. 17.4%, respectively, p = 0.012) ( Figure 1B).

Our survey revealed different opinions about the Austrian organ donation law depending on gender and level of information as well as hospital affiliation. Female nurses showed a trend toward negative opinion of the law, though their attitude improved with the level of information. However, informed staff seemed to be more willing to “override” the opt-out law by considering the wishes of the donor’s family. Nursing staff at University Hospital in Graz viewed the law more critically than the staff at Christian hospitals.

A possible explanation for the differences between the hospitals might be the Christian background. While the General Public Hospital of the Order of Saint Elisabeth and the hospitals of the Brothers of St. John of God expect from their staff to accept Christian values, the University in Hospital Graz imposes no such obligation. The Roman Catholic Church favors organ transplantation and sees the donation as an act of charity, [4] however, it has to be noted that the Roman Catholic Church is not unique in their acceptance of organ donation from brain-dead individuals. Although we did not ask for religiosity in particular, the differences between these two specific types of hospitals promote such speculations. We did not look at the diversity of religious belief or non-belief among nurses working at either the University Hospital or the Christian hospitals. We also did not study the impact of dominant Christian belief systems on the attitudes of nurses on organ donation law and practices.

Another explanation could be professional exposure to organ transplantation. At the Christian hospitals, ICU nurses care for organ donors, but only one of the seven hospitals actually performs (kidney) transplantations. In contrast, at the University Hospital in Graz, organ donations as well as heart, liver, pancreas and kidney implantations are routine procedures. In 2009, a survey on the attitude of healthcare professionals toward organ donation as part of the “Promotion of organ donation” project in Austria reported that physicians and other healthcare professionals find communication with relatives of the transplant patient psychologically stressful [5]. The frequency with which the nurses at University Hospital in Graz are confronted with such a situation is higher than in the other units and might contribute to the negative attitude of the nurses there. Furthermore, nursing staff in the Austrian Christian hospitals do not have to face common and serious complications like organ rejection and death of transplant patients. Kidney transplantations, which are performed in one of the Christian hospitals, generally entail fewer complications than e.g. liver or heart transplantations. Nurses at the University Hospital in Graz so are confronted more frequently with the downsides of organ donation, which might also have a negative influence their overall judgment of the law.

This survey did not cover psychological support for the ICU staff, for example supervision, coaching or psychotherapy sessions. Neither did we ask about the working atmosphere and solidarity among colleagues in the different intensive care units. Both aspects may have an influence on the attitude toward organ donation.

In conclusion, the existing organ donation law in Austria is well known among ICU healthcare professionals, though the groups surveyed vary in their support of the law. However, there seem to be differences between secular hospitals and religion-based hospitals. Further studies are necessary to find out why secular hospitals do worse in promoting positive attitudes to organ donation than religion-based hospitals. Also further in- depth studies on psychological aspects in hospitals where organ donors and organ recipients are treated could throw further light on these differences.

## Competing interests

The authors declare that they have no competing interests.

## Authors’ contributions

GZ analysed the data and wrote the manuscript, AH analysed the data and wrote the manuscript, EV performed the study and analysed the data, CA performed the study and reviewed the manuscript, MZ performed the study and reviewed the manuscript, PS performed the study, collected and analysed the data and reviewed the manuscript, VS designed the study, analysed the data and wrote the paper. All authors approved the final manuscript.

## Pre-publication history

The pre-publication history for this paper can be accessed here:

http://www.biomedcentral.com/1472-6939/15/46/prepub

## Supplementary Material

Additional file 1 Online survey announcement. Click here for file
